# Effects of Concurrent Training on Resuscitation and Cognitive Performance in Paramedics—A Pilot Study

**DOI:** 10.3390/healthcare12161599

**Published:** 2024-08-12

**Authors:** Tom Brandt, Andrea Schittenhelm, Daniel Kuhn Botelho, Tim Müller, Annette Schmidt

**Affiliations:** 1 Institute of Sports Science, University of the Bundeswehr Munich, 85579 Neubiberg, Germany; 2 NextGenerationEU, dtec.bw Project Smart Health Lab, Faculty of Human Sciences, Institute of Sports Science, Chair of Sport Biology, University of the Bundeswehr Munich, 85579 Neubiberg, Germany

**Keywords:** exercise, emergency, first responder, resuscitation quality, reaction time, working memory, divided attention, heart rate variability, ventilation, compression frequency

## Abstract

Paramedics work under physically and cognitively demanding conditions to provide emergency care. As physical fitness could positively impact the quality of patient care, we investigated within a pilot study whether concurrent training (CT) affects work-related performance parameters in paramedics. At baseline (T1), 16 paramedics performed cardiopulmonary resuscitation whereby resuscitation performance (RP; total resuscitation quality, compressions with correct frequency, and correct ventilation), cognitive performance (CP; reaction time, divided attention, and working memory), and heart rate variability (HRV) were assessed (pre-exertion). Then, participants climbed seven floors carrying 20 kg of gear before completing the same assessments again (post-exertion). The baseline testing was followed by a CT intervention (12 weeks, three sessions/week). After the intervention (T2), the two-stage testing was repeated. We analyzed whether the pre-exertion and post-exertion values, as well as the difference between the pre-exertion and post-exertion values, changed from T1 to T2. Nine paramedics (male: N = 7; age = 26.3 (SD = 8.17) years) took part in the study. The comparison of the pre-exertion values showed significantly better reaction times (*p* = 0.001) and divided attention (*p* = 0.02) and a trend toward greater working memory and RP parameters at T2. Regarding the post-exertion values, significant improvements in working memory (*p* = 0.03) and a trend toward improved reaction time, divided attention, and RP occurred at T2. The difference between the pre- and post-exertion values did not change for any parameter from T1 to T2. HRV decreased significantly from pre- to post-exertion (T1: *p* = 0.01, T2: *p* = 0.01). These results indicate that CT is a promising training concept to improve RP and CP in paramedics and should therefore be investigated further to increase patient care quality.

## 1. Introduction

Paramedics provide urgent care to patients under highly stressful conditions involving cognitive, psychomotor, and physical demands [1]. A common scenario for paramedics could involve carrying heavy equipment up several floors prior to the actual patient care. The patient care then comprises tasks like the loading/unloading or raising/lowering of stretchers, as well as patient handling. According to Fischer et al., paramedics have to handle loads of up to 89.9 kilograms (kg), which could accumulate to more than 350 kg to be lifted during a single emergency call [2]. In this regard, it should be noted that actual medical treatments such as cardiopulmonary resuscitation (CPR) are also physically and cognitively demanding.

Previous research found that already during the first 2 minutes (min) of CPR chest compression, the force already drops by 50–60 Newtons [3,4]. McDonald et al. further found that 79% of study participants reported that fatigue affected their CPR performance. Almost half of the decline in correctly delivered chest compressions again occurred between the 1st and 2nd min of CPR [5]. However, in the cited studies, participants performed CPR in a recovered state. In real-life scenarios as described above, it could be assumed that paramedics start medical treatments like CPR already pre-exhausted due to physical or mental stress.

Consequently, the impact of fatigue may be even greater than described in the mentioned studies, leading to detrimental effects on both the medical care of patients and the health of paramedics. For instance, the mismatch between the demands of paramedics’ duties and the physical fitness of paramedics is reflected in high rates of musculoskeletal disorders [1]. Additionally, paramedics are confronted with stressors such as trauma, shift work, and life-and-death situations. Accordingly, Kahn et al. reported that paramedics suffer from high levels of depression, anxiety, insomnia symptoms, fatigue, narcolepsy, and poorer sleep and well-being, which could negatively affect their cognitive function [6].

A method that is applied to investigate the physiological response to stressors is the measurement of heart rate variability (HRV) [7]. In previous research, the HRV response was analyzed in the context of different physically and mentally demanding tasks, including endurance, resistance, and mobility exercise, as well as cognitive tasks [7,8,9,10,11]. Wearables represent an opportunity to assess HRV in a non-invasive, non-intrusive way during real-world scenarios [7,12]. It was shown that even short-term HRV recordings of less than 2 min can be used for the assessment [13]. An HRV metric that can be used for short-term recordings as low as 50 seconds (s) to distinguish between stress and non-stress conditions is the root mean square of successive RR interval differences (RMSSD) [7].

While shift work and life-and-death situations can rarely be avoided, the perception and impact of physical incapacity may be reduced through physical training, thereby improving the quality of medical care for patients and the health of paramedics. Alongside physical fitness, cognitive function could also be improved by physical training. According to Logan et al., athletes outperformed non-athlete control groups in cognitive tasks like attention allocation and cognitive flexibility [14]. Previous research has suggested that paramedics should have the opportunity to improve their physical fitness through the implementation of guided, targeted exercise programs [1].

Based on the versatile physical demands placed on paramedics (e.g., cardiorespiratory fitness, strength), concurrent training (CT), i.e., the combination of resistance training with endurance exercise, could be an effective approach to improve fitness in paramedics [2,15]. In Germany, compulsory CT programs for paramedics have not been envisaged yet. However, examining the effectiveness of such programs in terms of medical care quality could have implications for practice and policy makers. As there is a lack of research in this field, small-scale studies could be conducted as a first step to evaluate methods, procedures, and possible outcomes that could be applicable to larger-scale studies. Therefore, it was investigated within a pilot study how a 12-week CT intervention impacts work-related performance parameters such as resuscitation performance (RP), cognitive performance (CP), and heart rate variability (HRV) in paramedics in pre- and post-exertion conditions.

## 2. Materials and Methods

### 2.1. Trial Oversight

This pilot study was conducted at the University of the Bundeswehr Munich (UniBw M). Participants attended 2 test sessions, before (T1) and after (T2) a 12-week CT intervention. Each session included two-stage testing which consisted of a pre-exertion and a post-exertion assessment (pre-exertion before the intervention = T1.1, post-exertion before the intervention = T1.2, pre-exertion after the intervention = T2.1, and post-exertion after the intervention = T2.2). During the CT intervention phase, participants performed 3 training sessions per week. The training sessions lasted 45–60 min and combined strength with endurance training.

**Figure 1:**
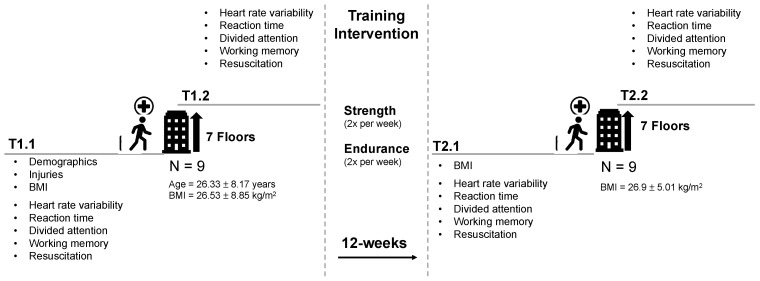
Study design. Abbreviations: BMI = body mass index; T1.1 = pre-exertion before intervention; T1.2 = post-exertion before intervention; T2.1 = pre-exertion after intervention; T2.2 = post-exertion after intervention.

The Institutional Ethics Committee of the UniBw M approved the study protocol, ensuring that it conformed to the ethical guidelines of the 1975 Declaration of Helsinki. Informed consent was obtained from all subjects involved in the study (6 February 2023; EK UniBw M 23-13). An overview of the study design is displayed in Figure 1.

### 2.2. Participants

This study involved healthy adults who were at least 18 years old and active as volunteer paramedics or first responders. In total, 16 participants signed up for the study, of which 7 (44%) discontinued the study. Of the 9 participants who took part in the study, 2 (22%) were female and 7 (78%) male. Their age ranged from 20 to 45 years. On average, the participants were 26.3 (standard deviation (SD) = 8.17) years of age, 179.11 (SD = 10.18) centimeters (cm) tall, and weighed 86.34 (SD = 24.58) kg.

### 2.3. Protocol and Outcomes

All tests were conducted by the same personnel throughout the study. The test personnel and participants were not blinded. Participants were asked to avoid any intense physical activity 24 h before the test sessions. They were further instructed to maintain the same fluid and food intake on the test days. Before the testing, participants sat down to answer questions regarding their age, gender, and cardiovascular or musculoskeletal disabilities that could prevent them from attending the testing or intervention. Then, their body mass and height were measured.

The actual testing consisted of a pre- and post-exertion assessment divided by a stair run. At first, heart rate variability (HRV) and cognitive performance (CP) were measured. Afterwards, participants performed CPR using a Resusci-Anne V (Laerdal Medical GmbH, Puchheim, Germany) while resuscitation performance (RP) was assessed. Then, participants had to climb 7 floors in their paramedic uniform while carrying a rescue backpack (16.9 kg) and an oxygen bag (3.1 kg). Immediately after arrival on the 7th floor, RP, CP, and HRV were measured in reversed order. This baseline two-stage testing was followed by the 12-week CT program. After completing the training program, participants were tested again using the same two-stage testing protocol. Consequently, each CP and RP parameter, as well as HRV, was measured at 4 time points.

To investigate how these parameters changed after climbing 7 floors in gear and to determine the effects of the CT program, the following comparisons were made: (a) T1.1 versus T1.2, (b) T2.1 versus T2.2, (c) T1.2–T1.1 versus T2.2–T2.1, (d) T1.1 versus T2.1, and (e) T1.2 versus T2.2.

In the first step, for T1 (a) and T2 (b), it was analyzed whether RP, CP, and HRV changed from the pre- to post-exertion measurements and whether exertion influenced these parameters. Two different approaches were chosen to estimate the possible effects of the CT intervention on RP, CP, and HRV. The first approach focused on the difference in the change from the pre- to post-exertion values between T1 and T2 (c). This was carried out to examine if the CT intervention influenced the exertion-induced changes in performance. The second approach was used to analyze whether the pre-exertion values before the intervention differed from those after the intervention (d). The same procedure was applied for the post-exertion values (e). The aim of the second approach (d, e) was to determine if the absolute performance improved after the intervention phase.

#### 2.3.1. Body Mass and Height

Body mass was measured with a SECA^®^ mBCA 515 scale (seca GmbH & Co., KG, Hamburg, Germany). Height was measured with a SECA^®^ 213 stadiometer (seca GmbH & Co., KG, Hamburg, Germany).

#### 2.3.2. Resuscitation Performance

For the RP testing, a Resusci-Anne V (Laerdal Medical GmbH, Puchheim, Germany) was used. The testing consisted of checking vital signs (responsiveness, breathing, pulse, etc.), making an emergency call, intubating, and resuscitating in a 30:2 rhythm for 150 seconds (s). The resuscitation data were recorded with a SimPad Plus (Laerdal Medical GmbH, Puchheim, Germany). RP was evaluated based on 3 scores: the percentage of compressions with the correct frequency, the percentage of correct ventilations, and total resuscitation quality.

#### 2.3.3. Cognitive Performance

CP was assessed with the MS Cognition app (interActive Systems GmbH, Berlin, Germany) which was developed by the associations “German Multiple Sclerosis Society” and “Action for People with Multiple Sclerosis”. It allows for the assessment of cognitive function in patients with multiple sclerosis, but also in healthy individuals. The testing consisted of 3 tasks to measure reaction time, divided attention, and working memory. Reaction time was measured with a matching-response test and given in milliseconds (ms). For this test, participants had to decide as fast as possible if the presented figures on the left side of the screen matched the figures on the right side. Therefore, they had to press the right (correct) or left (incorrect) key on a tablet screen. In a click-by-prompt task, participants had to select figures that matched a given verbal prompt by clicking on them. In the recall-2-back test, participants were shown a series of figures and had to decide if the current figure matched the figure shown two steps earlier. Depending on the performance in the click-by-prompt and recall-2-back tests, the MS Cognition app calculated a score for divided attention and working memory, with a higher score indicating better performance.

#### 2.3.4. Heart Rate Variability

For the heart rate measurement, a Polar H10 sensor and the Polar Flow app were used (Polar Electro GmbH, Büttelborn, Germany). The collected data were imported into Kubios HRV software (Kubios Oy, Kuopio, Finland) to calculate the RMSSD in ms. The RMSSD reflects the beat-to-beat variance in heart rate [16,17]. HRV was measured under two conditions during each test sessions to obtain the pre- and post-exertion HRV. The pre-exertion HRV measurement was performed in advance of the CP and RP testing. In contrast, the post-exertion HRV measurement was conducted directly after the RP and CP testing. A standardized measurement duration of 90 s in a seated position was defined.

### 2.4. Training Intervention

The intervention comprised a 12-week CT plan and was divided into 3 mesocycles, each lasting 4 weeks. Participants attended 3 training sessions per week with 48–72 h of rest between sessions. Of these 3 sessions, there was one strength- and one endurance-focused session as well as one session combining strength and endurance training. The combined sessions were 12 min longer than the pure strength sessions due to the sprint interval training following the strength training.

The strength training was based on compound movements. These were squats, deadlifts, bench presses, pull-ups, and shoulder presses. If participants were not able to perform the training in the gym, they were provided an alternative session consisting of bodyweight exercises including squats, front squats, push-ups, supermans, dive bombers, and burpees. Participants performed each exercise for 3–4 sets with 8–12 repetitions per exercise. The first training session was used for familiarization with the exercises and to determine the training weights for the set number of repetitions. Therefore, the participants worked up to the repetition maximum for the given number of repetitions within the defined number of sets (e.g., 8 repetitions and 4 sets for deadlifts). The load they achieved in the last set was then used as the training load for the following training session. Participants increased the load once they were able to perform all sets with the prescribed number of repetitions. The increments were 2.5 kg for upper-body and 5 kg for lower-body exercises. The strength training further included a 10 min high-intensity cycle. During these workouts, 2–3 compound movements had to be performed consecutively for 10–20 repetitions as fast as possible.

The endurance training consisted of high-intensity interval training and a long slow distance run session. In the high-intensity interval sessions, participants performed six sprint intervals. The participants were instructed to run at submaximal speeds, reaching 80–90% of their maximum velocity, during the sprints. The duration of work and rest for the sprint intervals was prescribed. The number of intervals was then increased by 1–2 intervals per week throughout the mesocycle. From mesocycle to mesocycle, the length of the sprints was increased while the resting periods were shortened. Regarding the long slow distance training, the runs during the first 2 mesocycles were performed at 60–70% and the third at 70–80% of the maximum heart rate. The training heart rate was calculated based on the estimated maximum heart rate (calculates as 220−age in years). Participants were allowed to train with their private heart rate sensors if they preferred to use them. Otherwise, a Polar H10 was provided. The target duration per run was 45 min in the 1st mesocycle, 60 min in the 2nd, and 45 min in the 3rd. The participants were instructed to record their training in a training log. The training program is provided in Supplementary Table S1.

### 2.5. Statistical Analysis

The statistical analysis was conducted with Microsoft Excel 2019 (Microsoft, Redmond, WA, USA) and SPSS 29^®^ (IBM SPSS, Armonk, NY, USA). The normality of the distributions was analyzed via Q-Q-plots and Kolmogorov–Smirnov tests. Changes between the pre- and post-exertion measurements at T1 (a) and T2 (b) were analyzed with a paired *t*-test if a normal distribution was shown. Otherwise, the Wilcoxon signed rank test was conducted. The same statistical approach was applied to test the differences between pre-exertion values at T1 and T2 (d), as well as post-exertion values at T1 and T2 (e). Furthermore, the differences between the pre- and post-exertion values for T1 and T2 (c) were analyzed via a paired *t*-test if the data were normally distributed. A Wilcoxon signed rank test was conducted when normality was not satisfied. Statistical significance was set at *p* ≤ 0.05. Statistical adjustments for multiple testing were not conducted. When the *t*-test was used, the effect size was given as Cohen’s d and interpreted as small (0.2), medium (0.5), or large (0.8) [18]. The effect size r was calculated based on the z-value and the sample size if the Wilcoxon signed rank test was applied. It was interpreted as small (0.1), medium (0.3), or large (0.5) [18]. The data are presented as mean (standard deviation).

## 3. Results

### 3.1. Resuscitation Performance

Total resuscitation quality was higher in the pre-exertion condition compared to the post-exertion condition before and after the CT intervention phase, but did not reach statistical significance (T1 *p* = 0.44, r = 0.26; T2 *p* = 0.48, r = 0.24). Similarly, participants had higher values for compressions with correct frequency in the pre-exertion condition, again without reaching statistical significance (T1 *p* = 0.87, r = 0.06; T2 *p* = 0.14, r = 0.49). In contrast, increased values were shown by the participants for correct ventilation post-exertion when compared to pre-exertion at T1 and T2. Nevertheless, the difference was not significant (T1 *p* = 0.7, d = 0.15; T2 *p* = 0.82, d = 0.08) (Table 1).

The change from pre- to post-exertion did not differ significantly between T1 and T2 for any of the RP parameters (total resuscitation: *p* = 0.88, d = 0.05; compressions with correct frequency: *p* = 0.16, r = 0.5; correct ventilation: *p* = 0.69, d = 0.15) (Table 2).

The comparison of the pre-exertion values before the CT intervention with those after the CT intervention indicated that all three RP parameters were higher after the intervention phase. Nevertheless, neither total resuscitation (*p* = 0.21, r = 0.42) nor compressions with correct frequency (*p* = 0.4, r = 0.28) nor correct ventilation (*p* = 0.06, d = 0.8) changed significantly. The comparison of post-exertion measures revealed greater, but non-significant, performance for total resuscitation (*p* = 0.72, r = 0.12) and correct ventilation (*p* = 0.18, d = 0.13) at T2, whereas performance for compressions with correct frequency was lower, but again, not significantly (*p* = 0.67, d = 0.15). (Table 3).

### 3.2. Cognitive Performance

**Figure 2:**
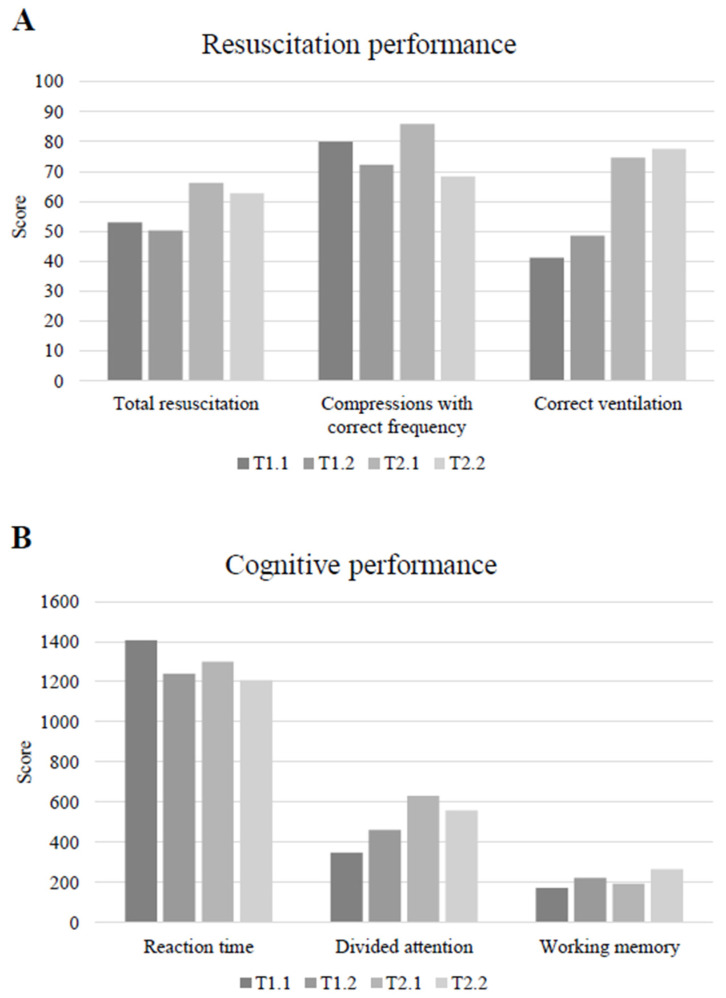
The resuscitation (**A**) and cognitive performance (**B**) of the participants (N = 9) before and after the intervention phase during the pre- and post-exertion conditions. Abbreviations: T1.1 = pre-exertion before intervention; T1.2 = post-exertion before intervention; T2.1 = pre-exertion after intervention; T2.2 = post-exertion after intervention.

Reaction time decreased significantly between the pre- and the post-exertion conditions at T1 and T2 (T1 *p* < 0.001, d = 1.82; T2 *p* = 0.002, d = 1.48). A significant improvement occurred for divided attention from pre- to post-exertion at T1 (*p* = 0.02, r = 0.77), but not at T2 (*p* = 0.44, r = 0.26). Working memory scores improved from pre- to post-exertion at T1 (*p* = 0.001, d = 1.6) and T2 (*p* < 0.001, d = 1.86) (Table 1). The change from pre- to post-exertion CP did not differ significantly between T1 and T2 (reaction time: *p* = 0.11, d = 61; divided attention: *p* = 0.12, d = 0.59; working memory: *p* = 0.15, d = 0.54) (Table 2). In this respect, it is important to note that participants had significantly better reaction times (*p* = 0.001, d = 1.64) and divided attention (*p* = 0.02, r = 0.77) in the pre-exertion measurement at T2 compared to T1, whereas improvements in working memory did not reach statistical significance (*p* = 0.21, d = 0.46). The comparison of the post-exertion values revealed a similar trend, with differences only being significant for working memory (*p* = 0.03, d = 0.85), but not for reaction time (*p* = 0.45, d = 0.27) and divided attention (*p* = 0.09, r = 0.57). (Table 3). The RP and CP parameters before and after the intervention phase are displayed in Figure 2.

### 3.3. Heart Rate Variability

HRV decreased significantly from the pre-exertion condition to the post-exertion condition before and after the intervention phase (T1 *p* = 0.01, r = 0.89; T2 *p* = 0.01, r = 0.89) (Table 1). The change in HRV from pre- to post-exertion was not significantly different between T1 and T2 (*p* = 0.15, d = 0.54) (Table 2). Although not significant, it should be noted that the participants showed lower pre-exertion HRV at T2 compared to T1 (*p* = 0.11, r = 0.53). A minor non-significant difference was found for post-exertion HRV (*p* = 0.86, r = 0.06) (Table 3).

## 4. Discussion

In this pilot study, it was investigated how 12 weeks of CT impacts the work-related performance parameters of paramedics during resuscitation scenarios. It was of particular interest whether CT would affect drops in work-related performance associated with physical exertion. Therefore, it was analyzed whether the induced physical stress had an effect on the participants’ performance. With regard to RP, no significant differences were found between the pre- and post-exertion conditions at either T1 or T2. Nevertheless, a negative trend was observed regarding total resuscitation quality and compressions with the correct frequency before and after the CT intervention with small to medium effect sizes. This observation is in line with previous studies, as a negative impact of exertion on resuscitation performance has already been confirmed [3,4,5]. In contrast, a positive trend with regard to correct ventilation was seen. This finding suggests that RP may need to be evaluated in a more differentiated way in future studies since the influence of stress may vary depending on the specific subtask. This was also evident in the CP results. For reaction time and working memory, significant improvements at T1 and T2 with large effect sizes were found. A significant improvement also occurred for divided attention at T1, whereas the drop in performance at T2 did not reach statistical significance.

That participants were affected by the induced stressors was reflected by significant reductions in HRV from pre- to post-exertion measurements at T1 and T2. The decrease in HRV indicated increased sympathetic action preparing the participants for the performed tasks [19,20,21]. It is possible that the induced stress was just at the right level for the participants to display greater performance when compared to the pre-exertion condition. This is in line with the results of Murray et al., who found that a moderate level of arousal post-exercise was associated with improved cognitive performance [11]. Similar results were obtained in previous studies with respect to reaction times [22]. However, it is important to note that HRV—as a physiological measure—is not only influenced by physiological but also by psychological stress [8,20,23]. Thus, to what extent HRV was influenced by physiological or psychological stressors remains unclear and could have differed between individuals. This should be considered carefully in future studies, when HRV is measured in conjunction with physically demanding tasks [8]. In fact, both stressful tasks and their mere anticipation can lead to a reduction in HRV [24]. This might explain why the participants showed HRV values that tended to be in the upper range of the norm for this age group at T1 (59.22 (34.52) ms), whereas at T2 (42.67 (20.35) ms), HRV was further towards the lower end of the scale [25,26]. In the present study, participants knew that the intervention was intended to improve their performance. It is therefore possible that they attuned themselves to the upcoming tests in order to beat their pre-CT intervention performance. Souza et al. described a similar phenomenon in athletes who also displayed reduced HRV values before competitions [21]. Indeed, the participants showed both better RP and CP after the intervention phase in the pre-exertion condition, with significant improvements in reaction time and divided attention. In terms of resuscitation, as it was a physically demanding task, the participants’ performance may have benefited from the fitness-enhancing effects of CT [3,4,5]. This was supported by the observed heart rate values. Although the physical stress before and after the intervention phase did not change objectively, the participants showed significantly lower average heart rates during the scenarios at T2 (130 (19.09) bpm) compared to T1 (144 (17.06) bpm) indicating lower relative physical demands with respect to fatigue (*p* = 0.02, r = 1.03).

While less fatigue should have aided RP, it must not be ignored that the technical proficient execution of cardiovascular resuscitation is also cognitively demanding [27]. Since physical activity and fitness are associated with improved cognitive function, greater RP could also be influenced by improved cognition [28,29]. Significant improvements in reaction time (*p* = 0.001, d = 1.64) and divided attention (*p* = 0.02, r = 0.77) and a trend toward greater working memory (*p* = 0.21, d = 0.46) after the intervention phase support this assumption.

As described above, the induced stress influenced the RP, CP, and HRV of the participants before and after the intervention phase. Looking at the differences from the pre- to post-exertion measurements, there was a trend of less adverse effects before the intervention phase. Yet, the differences in the change between T1 and T2 were not significant for any parameter. Regardless of the extent to which RP and CP changed due to exertion, the absolute quality with which paramedics actually resuscitate patients under stress remains of interest in a real resuscitation scenario. Despite slightly greater declines in RP and CP between the pre- and post-exertion measurements at T2, participants still displayed greater absolute performance values in every assessed parameter, except for compressions with correct frequency, when compared to T1. In this context, it is noteworthy that HRV decreased less from the pre- to post-exertion measurement at T2 (26.44 (15.27) ms) compared to T1 (41.67 (34.55) ms) and thereby reached a similar level at both post-exertion measurements (T1: 17.56 (12.5) ms, T2: 16.22 (12.19) ms). Consequently, participants performed better after the intervention phase, without significant differences in the objectively measured stress levels. From this point of view, it could be deduced that the intervention had a positive effect as the patients would have received better-quality resuscitation. This could ultimately lead to fewer medical complications and higher chances of survival.

Nevertheless, when interpreting the results, several limitations need to be considered. Because this was a pilot study, the number of participants was relatively low. Accordingly, for some parameters, even medium to large effects did not reach statistical significance. Therefore, to verify the results of the current study, a larger sample size is suggested. In order to achieve a power of at least 80% with a one-sided 5% significance level, a sample size of 30 to 50 participants would be required for most parameters if the same study design was used and the dropout was similar. Contrastingly, a sample size of N = 2475 would be needed for the smallest effect found in this study (difference in change in resuscitation performance; T1.2–T1.1 versus T2.1–T2.2; d = 0.05). This indicates that the design must be modified if this parameter is investigated to reduce the required sample size. In this respect, the duration of the training intervention could be extended or the physical and psychological stress generated by the resuscitation scenario might be further increased. Introducing a control group that does not perform a prescribed training program would be another option. This could also help in determining to what extent the effects could be explained by the CT intervention. For instance, in the present study, the lack of a control group meant that practice effects were not adequately controlled. This factor is particularly important with respect to the CP testing. Since the participants were experienced paramedics who were familiar with cardiovascular resuscitation, it can be assumed that practice effects with regard to RP were less likely. Nevertheless, including a familiarization session for all conducted tests to minimize the influence of practice on the outcome variables is recommended. It should also be considered whether, for studies focusing on specific aspects of CP, other psychological diagnostic tests that were explicitly designed and validated for the construct of interest might be superior. For example, the Vienna test system offers a comprehensive selection of tests and could be applicable to investigations conducted in a laboratory setting [30]. In order to better understand the causal relationship between the CT intervention and the outcomes, follow-up studies should assess whether fitness (e.g., endurance and strength) improved after the intervention. Furthermore, female participants were under-represented in this study based on the demographic characteristics among paramedics [31]. This can be explained by the fact that the majority of students at the UniBw M were male. Future studies should take this aspect into account, especially if they are conducted at sites with a similar gender distribution. In future larger-scale studies with intervention and control groups, it is further recommended to apply a double-blinded design. Assessments of satisfaction with the intervention, the likelihood of recommending CT to other paramedics, and detailed reasons for dropouts in subsequent studies would also help to tailor training to the needs of paramedics.

## 5. Conclusions

In conclusion, it was shown that stress leads to divergent changes in the performance of paramedics in resuscitation scenarios, with RP tending to decrease and CP to improve under stress. Although stress-related negative effects on RP were not reduced due to the intervention, the overall improvement in RP and CP after the intervention phase indicated that CT may have had positive effects. CT thus appears to be a promising training intervention for paramedics, but its effects need verification through further research.

## Figures and Tables

**Table 1:** Change from pre- and post-exertion values before (T1.1 versus T1.2) and after (T2.1 versus 2.2) the intervention phase.

	T1.1	T1.2	*p*	d	r	T2.1	T2.2	*p*	d	r
Total resuscitation (%)	52.9 (31.83)	50.22 (33.2)	0.44		0.26	66.11 (18.05)	62.67 (16.27)	0.48		0.24
Compressions with correct frequency (%)	79.9 (31.97)	72.22 (38.8)	0.87		0.06	85.9 (21.34)	68.33 (32.19)	0.14		0.49
Correct ventilation (%)	41.13 (40.2)	48.5 (44.8)	0.7	0.15		74.67 (24.38)	77.56 (27.15)	0.82	0.08	
Reaction time (ms)	1406.11 (164)	1239.56 (114.44)	<0.001	1.82		1298.33 (155.63)	1205.56 (199.48)	0.002	1.48	
Divided attention (score)	347 (71.61)	460.44 (148.91)	0.02		0.77	631.11 (373.82)	557.44 (223.26)	0.44		0.26
Working memory (score)	171.44 (75.13)	219.44 (65.32)	0.001	1.6		192.11 (86.57)	266.44 (82.68)	<0.001	1.86	
HRV (ms)	59.22 (34.52)	17.56 (12.5)	0.01		0.89	42.67 (20.35)	16.22 (12.19)	0.01		0.89

Note: Values at T1.1, T1.2, T2.1, and T2.2 are expressed as mean (standard deviation). Significance level was set at *p* < 0.05 for differences between T1.1 and T1.2 as well as T2.1 and T2.2. Effect size is given as Cohen’s d if *t*-test was applied. Pearson’s r is given if Wilcoxon test was conducted. Abbreviations: HRV = heart rate variability; ms = milliseconds; T1.1 = pre-exertion before intervention; T1.2 = post-exertion before intervention; T2.1 = pre-exertion after intervention; T2.2 = post-exertion after intervention.

**Table 2:** Differences in change from pre- to post-exertion measurements before and after intervention phase ((T1.2–T1.1) versus (T2.2–T2.1)).

	T1	T2	*p*	d	r
Total resuscitation (%)	2.67 (7.92)	3.44 (12.38)	0.88	0.05	
Compressions with correct frequency (%)	7.67 (34.78)	12.56 (21.38)	0.16		0.5
Correct ventilation (%)	7.38 (51.04)	2.5 (36.5)	0.69	0.15	
Reaction time (ms)	166.56 (91.56)	92.78 (62.79)	0.11	0.61	
Divided attention (score)	113.44 (129.25)	73.67 (315.55)	0.12	0.59	
Working memory (score)	48 (30.05)	74.33 (39.99)	0.15	0.54	
HRV (ms)	41.67 (34.55)	26.44 (15.27)	0.15	0.54	

Note: Values for differences at T1 and T2 are expressed as mean (standard deviation). Differences were calculated by subtracting T1.2 from T1.1 for T1 and T2.2 from T2.1 for T2. Significance level was set at *p* < 0.05. Effect size is given as Cohen’s d if *t*-test was applied. Pearson’s r is given if Wilcoxon test was conducted. Abbreviations: HRV = heart rate variability; ms = milliseconds; T1.1 = pre-exertion before intervention; T1.2 = post-exertion before intervention; T2.1 = pre-exertion after intervention; T2.2 = post-exertion after intervention.

**Table 3:** Differences between pre-exertion values before and after CT intervention (T1.1 versus T2.1) as well as post-exertion values before and after CT intervention (T1.2 versus T2.2).

	T1.1	T2.1	*p*	d	r	T1.2	T2.2	*p*	d	r
Total resuscitation (%)	52.9 (31.83)	66.11 (18.05)	0.21		0.42	50.22 (33.2)	62.67 (16.27)	0.72		0.12
Compressions with correct frequency (%)	79.9 (31.97)	85.9 (21.34)	0.4		0.28	72.22 (38.8)	68.33 (32.19)	0.67	0.15	
Correct ventilation (%)	41.13 (40.2)	74.67 (24.38)	0.06	0.80		48.5 (44.8)	77.56 (27.15)	0.18	0.13	
Reaction time (ms)	1406.11 (164)	1298.33 (155.63)	0.001	1.64		1239.56 (114.44)	1205.56 (199.48)	0.45	0.27	
Divided attention (score)	347 (71.61)	631.11 (373.82)	0.02		0.77	460.44 (148.91)	557.44 (223.26)	0.09		0.57
Working memory (score)	171.44 (75.13)	192.11 (86.57)	0.21	0.46		219.44 (65.32)	266.44 (82.68)	0.03	0.85	
HRV (ms)	59.22 (34.52)	42.67 (20.35)	0.11		0.53	17.56 (12.5)	16.22 (12.19)	0.86		0.06

Note: Values at T1.1, T1.2, T2.1, and T2.2 are expressed as mean (standard deviation). Significance level was set at *p* < 0.05 for differences between T1.1 and T2.1 as well as T1.2 and T2.1. Effect size is given as Cohen’s d if *t*-test was applied. Pearson’s r is given if Wilcoxon test was conducted. Abbreviations: HRV = heart rate variability; ms = milliseconds; T1.1 = pre-exertion before intervention; T1.2 = post-exertion before intervention; T2.1 = pre-exertion after intervention; T2.2 = post-exertion after intervention.

## Data Availability

The data that support the findings of this study are available from the corresponding author upon reasonable request.

## Supplementary Materials

The following supporting information can be downloaded at: https://www.mdpi.com/article/10.3390/healthcare12161599/s1. Table S1: Training template
