# Supplementary material for: Effects of Concurrent Training on Resuscitation and Cognitive Performance in Paramedics—A Pilot Study
Source: Healthcare (Basel). 2024 Aug 12;12(16):1599. doi: 10.3390/healthcare12161599 (PMC11353908; doi:10.3390/healthcare12161599)
Supplement: Supplementary file 1 [file healthcare-12-01599-s001.zip › healthcare-3131774-supplementary.pdf]

Supplemental table S1 Training template

**Week 1 - 4**	Training session 1			Training session 2			Training session 3
	Exercise	Repetitions	Sets	Exercise	Repetitions	Sets	
Strength	Deadlift<br>Back squat<br>Shoulder press<br>Bench press<br>Lat pull	8<br>12<br>8<br>12<br>10	4<br>3<br>5<br>3<br>3	Back squats<br>Deadlift<br>Bench press<br>Shoulder press<br>Lat pull	8<br>12<br>8<br>12<br>10	4<br>3<br>5<br>3<br>3	
High intensity strength cycle	5 rounds for time<br>Pull ups<br>Burpees<br>Squats	 10<br>15<br>20	5 rounds	10 min<br>Deadlift<br>Shoulder press	 10<br>10	As many rounds as possible	
Endurance	Run on track<br>3 min warm up run<br>6 intervals of 15 sec sprint and 45 sec rest<br>3 min cool down run						Run for 60 min at 60 – 70 % of maximum heart rate

**Week 5 - 8**	Training session 1			Training session 2			Training session 3
	Exercise	Repetitions	Sets	Exercise	Repetitions	Sets	
Strength	Deadlift<br>Back squat<br>Shoulder press<br>Bench press<br>Lat pull	8<br>12<br>8<br>12<br>10	4<br>3<br>5<br>3<br>3	Back squats<br>Deadlift<br>Bench press<br>Shoulder press<br>Lat pull	8<br>12<br>8<br>12<br>10	4<br>3<br>5<br>3<br>3	
High intensity strength cycle	7 rounds for time<br>Pull ups<br>Burpees<br>Squats	 10<br>15<br>20	7 rounds	10 min<br>Deadlift<br>Shoulder press	 15<br>15	As many rounds as possible	
Endurance	Run on track<br>3 min warm up run<br>6 intervals of 20 sec sprint and 40 sec rest<br>3 min cool down run						Run for 45 min at 60 – 70 % of maximum heart rate

**Week 9 - 12**	Training session 1			Training session 2			Training session 3		
	Exercise	Repetitions	Sets	Exercise	Repetitions	Sets			
Strength	Deadlift<br>Back squat<br>Shoulder press<br>Bench press<br>Lat pull	8<br>12<br>8<br>12<br>10	4<br>3<br>5<br>3<br>3	Back squats<br>Deadlift<br>Bench press<br>Shoulder press<br>Lat pull	8<br>12<br>8<br>12<br>10	4<br>3<br>5<br>3<br>3			
High intensity strength cycle	9 rounds for time<br>Pull ups<br>Burpees<br>Squats	 10<br>15<br>20	7 rounds	10 min<br>Deadlift<br>Shoulder press	 20<br>20	As many rounds as possible			
Endurance	Run on track<br>3 min warm up run<br>6 intervals of 30 sec sprint and 30 sec rest<br>3 min cool down run						Run for 45 min at 70 – 80 % of maximum heart rate		
